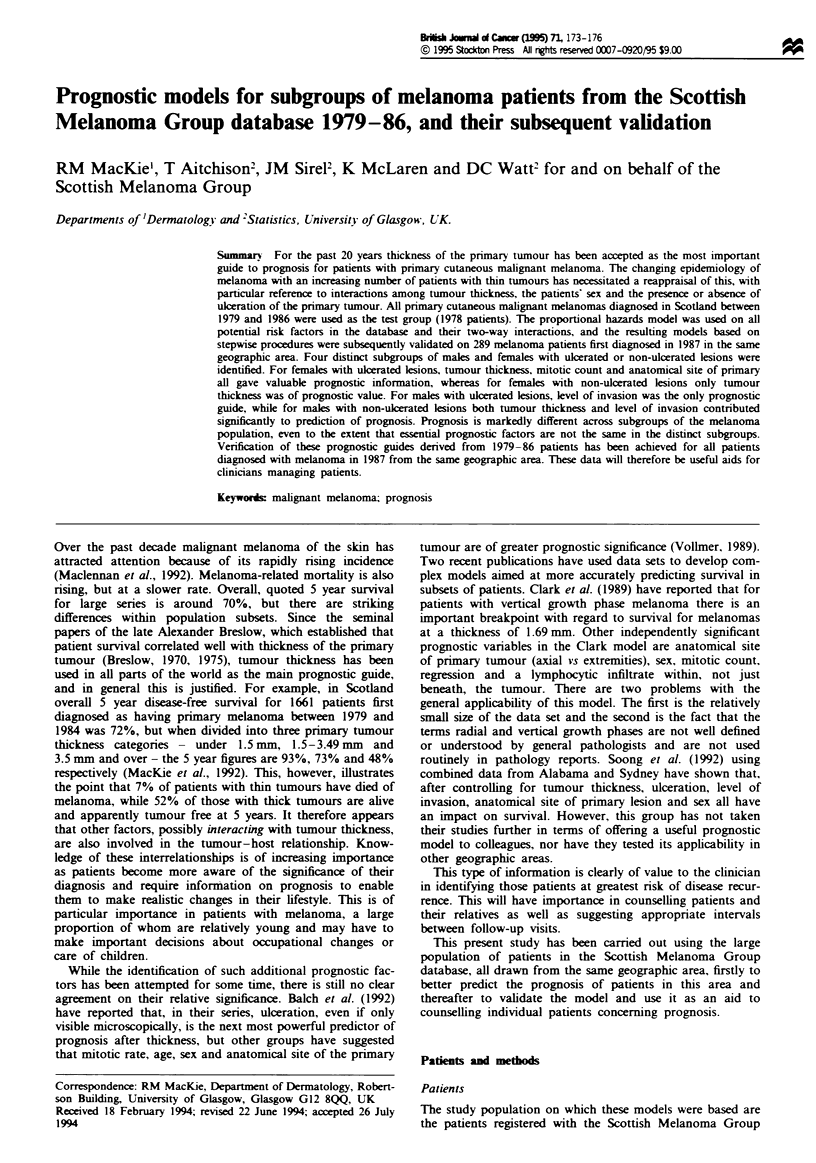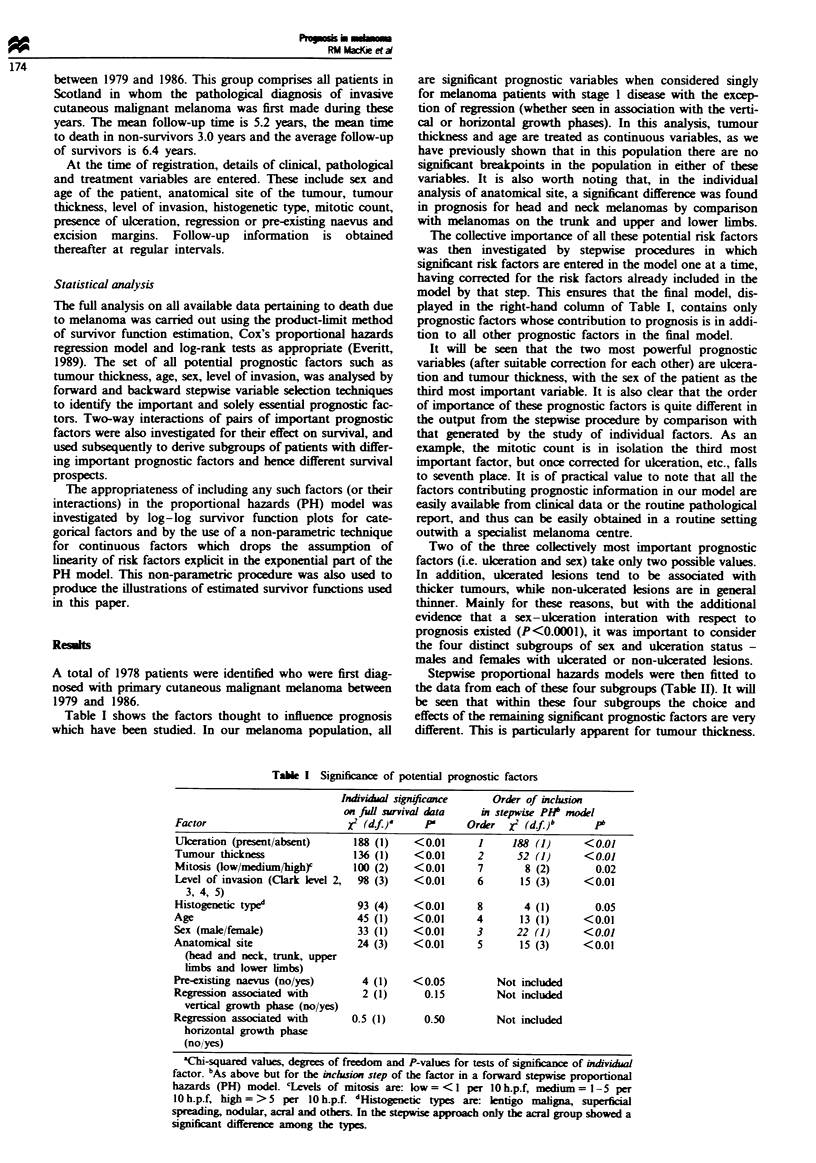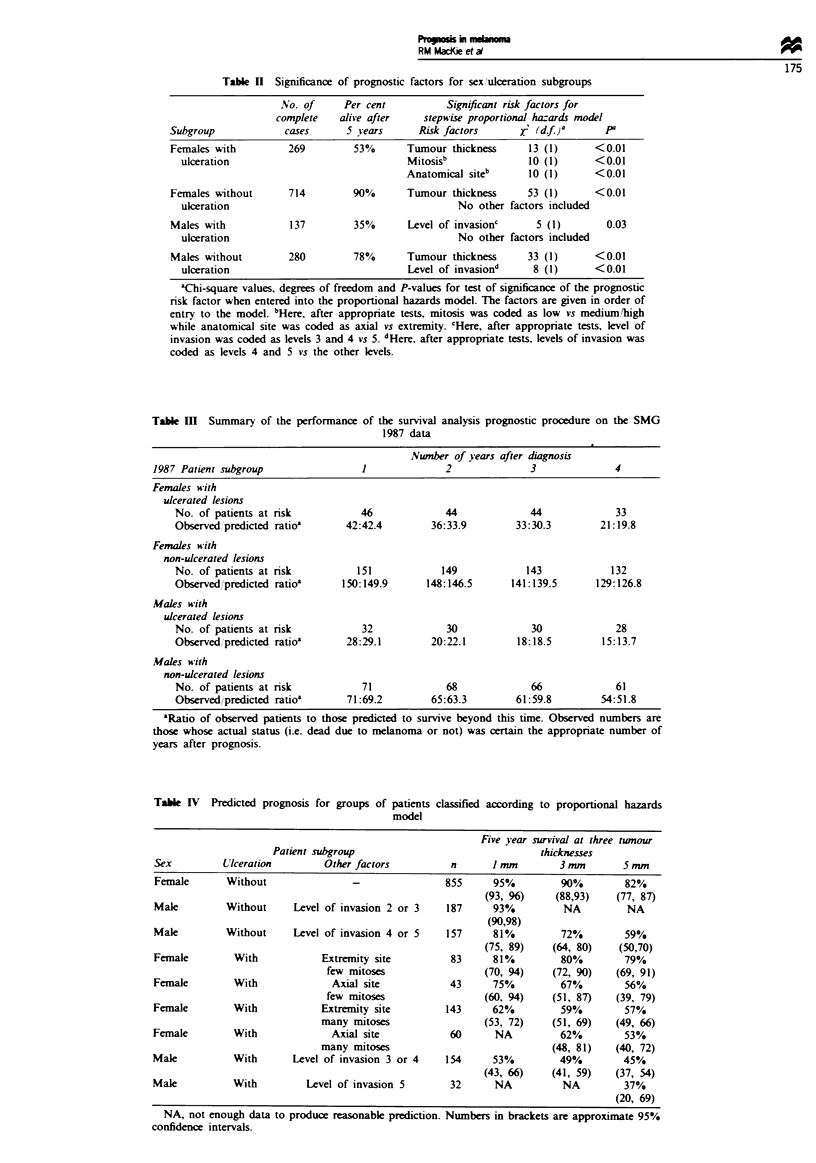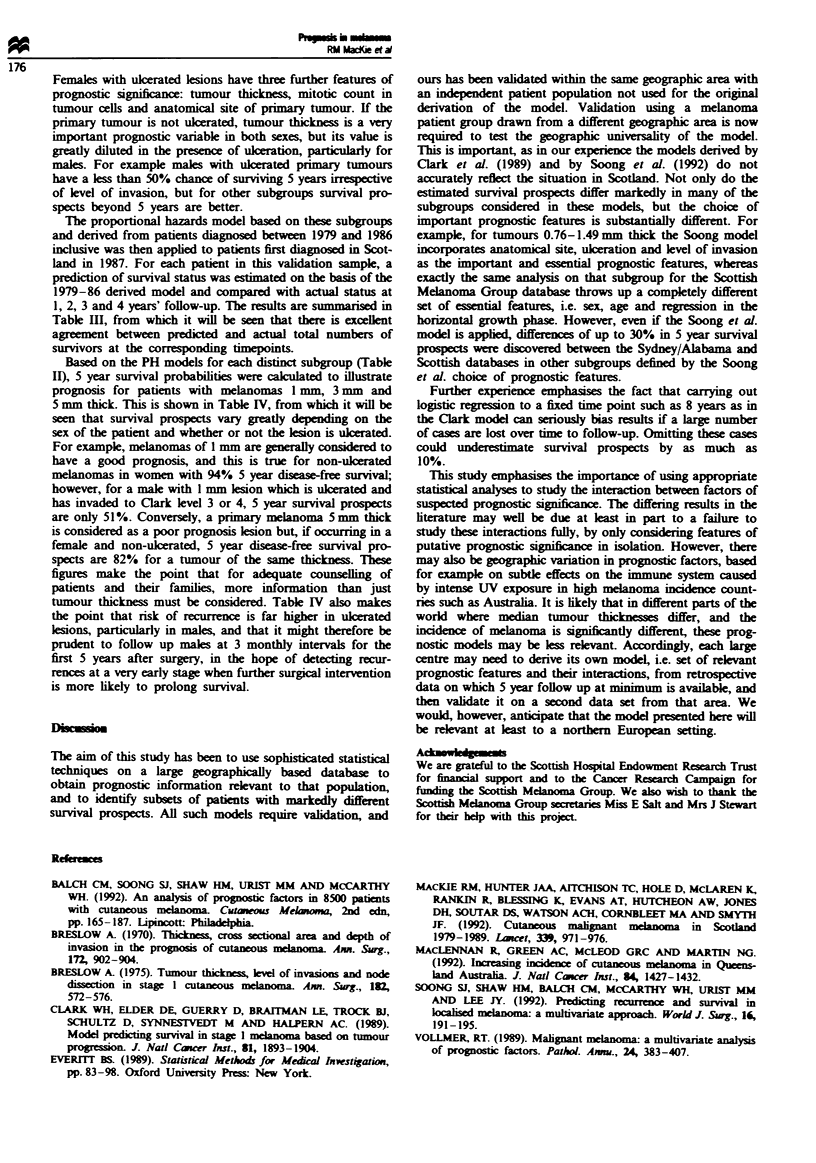# Prognostic models for subgroups of melanoma patients from the Scottish Melanoma Group database 1979-86, and their subsequent validation.

**DOI:** 10.1038/bjc.1995.35

**Published:** 1995-01

**Authors:** R. M. MacKie, T. Aitchison, J. M. Sirel, K. McLaren, D. C. Watt

**Affiliations:** Department of Dermatology, University of Glasgow, UK.

## Abstract

For the past 20 years thickness of the primary tumour has been accepted as the most important guide to prognosis for patients with primary cutaneous malignant melanoma. The changing epidemiology of melanoma with an increasing number of patients with thin tumours has necessitated a reappraisal of this, with particular reference to interactions among tumour thickness, the patients' sex and the presence or absence of ulceration of the primary tumour. All primary cutaneous malignant melanomas diagnosed in Scotland between 1979 and 1986 were used as the test group (1978 patients). The proportional hazards model was used on all potential risk factors in the database and their two-way interactions, and the resulting models based on stepwise procedures were subsequently validated on 289 melanoma patients first diagnosed in 1987 in the same geographic area. Four distinct subgroups of males and females with ulcerated or non-ulcerated lesions were identified. For females with ulcerated lesions, tumour thickness, mitotic count and anatomical site of primary all gave valuable prognostic information, whereas for females with non-ulcerated lesions only tumour thickness was of prognostic value. For males with ulcerated lesions, level of invasion was the only prognostic guide, while for males with non-ulcerated lesions both tumour thickness and level of invasion contributed significantly to prediction of prognosis. Prognosis markedly different across subgroups of the melanoma population, even to the extent that essential prognostic factors are not the same in the distinct subgroups. Verification of these prognostic guides derived from 1979-86 patients has been achieved for all patients diagnosed with melanoma in 1987 from the same geographic area. These data will therefore be useful aids for clinicians managing patients.


British Journal of Cancer (1995) 71, 173-176

© 1995 Stockton Press All rights reserved 0007-0920/95 $9.00

### Prognostic models for subgroups of melanoma patients from the Scottish Melanoma Group database 1979-86, and their subsequent validation

RM MacKie[1], T Aitchison[2], JM Sirel[2], K McLaren and DC Watt[2] for and on behalf of the Scottish Melanoma Group

*Departments of [1]Dermatology and [2]Statistics, University of Glasgow, UK.*

**Summary** For the past 20 years thickness of the primary tumour has been accepted as the most important guide to prognosis for patients with primary cutaneous malignant melanoma. The changing epidemiology of melanoma with an increasing number of patients with thin tumours has necessitated a reappraisal of this, with particular reference to interactions among tumour thickness, the patients' sex and the presence or absence of ulceration of the primary tumour. All primary cutaneous malignant melanomas diagnosed in Scotland between 1979 and 1986 were used as the test group (1978 patients). The proportional hazards model was used on all potential risk factors in the database and their two-way interactions, and the resulting models based on stepwise procedures were subsequently validated on 289 melanoma patients first diagnosed in 1987 in the same geographic area. Four distinct subgroups of males and females with ulcerated or non-ulcerated lesions were identified. For females with ulcerated lesions, tumour thickness, mitotic count and anatomical site of primary all gave valuable prognostic information, whereas for females with non-ulcerated lesions only tumour thickness was of prognostic value. For males with ulcerated lesions, level of invasion was the only prognostic guide, while for males with non-ulcerated lesions both tumour thickness and level of invasion contributed significantly to prediction of prognosis. Prognosis is markedly different across subgroups of the melanoma population, even to the extent that essential prognostic factors are not the same in the distinct subgroups. Verification of these prognostic guides derived from 1979-86 patients has been achieved for all patients diagnosed with melanoma in 1987 from the same geographic area. These data will therefore be useful aids for clinicians managing patients.

**Keywords:** malignant melanoma; prognosis

Over the past decade malignant melanoma of the skin has attracted attention because of its rapidly rising incidence (Maclennan *et al.*, 1992). Melanoma-related mortality is also rising, but at a slower rate. Overall, quoted 5 year survival for large series is around 70%, but there are striking differences within population subsets. Since the seminal papers of the late Alexander Breslow, which established that patient survival correlated well with thickness of the primary tumour (Breslow, 1970, 1975), tumour thickness has been used in all parts of the world as the main prognostic guide, and in general this is justified. For example, in Scotland overall 5 year disease-free survival for 1661 patients first diagnosed as having primary melanoma between 1979 and 1984 was 72%, but when divided into three primary tumour thickness categories - under 1.5 mm, 1.5-3.49 mm and 3.5 mm and over - the 5 year figures are 93%, 73% and 48% respectively (MacKie *et al.*, 1992). This, however, illustrates the point that 7% of patients with thin tumours have died of melanoma, while 52% of those with thick tumours are alive and apparently tumour free at 5 years. It therefore appears that other factors, possibly *interacting* with tumour thickness, are also involved in the tumour-host relationship. Knowledge of these interrelationships is of increasing importance as patients become more aware of the significance of their diagnosis and require information on prognosis to enable them to make realistic changes in their lifestyle. This is of particular importance in patients with melanoma, a large proportion of whom are relatively young and may have to make important decisions about occupational changes or care of children.

While the identification of such additional prognostic factors has been attempted for some time, there is still no clear agreement on their relative significance. Balch *et al.* (1992) have reported that, in their series, ulceration, even if only visible microscopically, is the next most powerful predictor of prognosis after thickness, but other groups have suggested that mitotic rate, age, sex and anatomical site of the primary tumour are of greater prognostic significance (Vollmer, 1989). Two recent publications have used data sets to develop complex models aimed at more accurately predicting survival in subsets of patients. Clark *et al.* (1989) have reported that for patients with vertical growth phase melanoma there is an important breakpoint with regard to survival for melanomas at a thickness of 1.69 mm. Other independently significant prognostic variables in the Clark model are anatomical site of primary tumour (axial *vs* extremities), sex, mitotic count, regression and a lymphocytic infiltrate within, not just beneath, the tumour. There are two problems with the general applicability of this model. The first is the relatively small size of the data set and the second is the fact that the terms radial and vertical growth phases are not well defined or understood by general pathologists and are not used routinely in pathology reports. Soong *et al.* (1992) using combined data from Alabama and Sydney have shown that, after controlling for tumour thickness, ulceration, level of invasion, anatomical site of primary lesion and sex all have an impact on survival. However, this group has not taken their studies further in terms of offering a useful prognostic model to colleagues, nor have they tested its applicability in other geographic areas.

This type of information is clearly of value to the clinician in identifying those patients at greatest risk of disease recurrence. This will have importance in counselling patients and their relatives as well as suggesting appropriate intervals between follow-up visits.

This present study has been carried out using the large population of patients in the Scottish Melanoma Group database, all drawn from the same geographic area, firstly to better predict the prognosis of patients in this area and thereafter to validate the model and use it as an aid to counselling individual patients concerning prognosis.

### Patients and methods

### *Patients*

The study population on which these models were based are the patients registered with the Scottish Melanoma Group

Correspondence: RM MacKie, Department of Dermatology, Robertson Building, University of Glasgow, Glasgow G12 8QQ, UK
Received 18 February 1994; revised 22 June 1994; accepted 26 July 1994

between 1979 and 1986. This group comprises all patients in Scotland in whom the pathological diagnosis of invasive cutaneous malignant melanoma was first made during these years. The mean follow-up time is 5.2 years, the mean time to death in non-survivors 3.0 years and the average follow-up of survivors is 6.4 years.

At the time of registration, details of clinical, pathological and treatment variables are entered. These include sex and age of the patient, anatomical site of the tumour, tumour thickness, level of invasion, histogenetic type, mitotic count, presence of ulceration, regression or pre-existing naevus and excision margins. Follow-up information is obtained thereafter at regular intervals.

### *Statistical analysis*

The full analysis on all available data pertaining to death due to melanoma was carried out using the product-limit method of survivor function estimation, Cox's proportional hazards regression model and log-rank tests as appropriate (Everitt, 1989). The set of all potential prognostic factors such as tumour thickness, age, sex, level of invasion, was analysed by forward and backward stepwise variable selection techniques to identify the important and solely essential prognostic factors. Two-way interactions of pairs of important prognostic factors were also investigated for their effect on survival, and used subsequently to derive subgroups of patients with differing important prognostic factors and hence different survival prospects.

The appropriateness of including any such factors (or their interactions) in the proportional hazards (PH) model was investigated by log–log survivor function plots for categorical factors and by the use of a non-parametric technique for continuous factors which drops the assumption of linearity of risk factors explicit in the exponential part of the PH model. This non-parametric procedure was also used to produce the illustrations of estimated survivor functions used in this paper.

### Results

A total of 1978 patients were identified who were first diagnosed with primary cutaneous malignant melanoma between 1979 and 1986.

Table I shows the factors thought to influence prognosis which have been studied. In our melanoma population, all are significant prognostic variables when considered singly for melanoma patients with stage 1 disease with the exception of regression (whether seen in association with the vertical or horizontal growth phases). In this analysis, tumour thickness and age are treated as continuous variables, as we have previously shown that in this population there are no significant breakpoints in the population in either of these variables. It is also worth noting that, in the individual analysis of anatomical site, a significant difference was found in prognosis for head and neck melanomas by comparison with melanomas on the trunk and upper and lower limbs.

The collective importance of all these potential risk factors was then investigated by stepwise procedures in which significant risk factors are entered in the model one at a time, having corrected for the risk factors already included in the model by that step. This ensures that the final model, displayed in the right-hand column of Table I, contains only prognostic factors whose contribution to prognosis is in addition to all other prognostic factors in the final model.

It will be seen that the two most powerful prognostic variables (after suitable correction for each other) are ulceration and tumour thickness, with the sex of the patient as the third most important variable. It is also clear that the order of importance of these prognostic factors is quite different in the output from the stepwise procedure by comparison with that generated by the study of individual factors. As an example, the mitotic count is in isolation the third most important factor, but once corrected for ulceration, etc., falls to seventh place. It is of practical value to note that all the factors contributing prognostic information in our model are easily available from clinical data or the routine pathological report, and thus can be easily obtained in a routine setting outwith a specialist melanoma centre.

Two of the three collectively most important prognostic factors (i.e. ulceration and sex) take only two possible values. In addition, ulcerated lesions tend to be associated with thicker tumours, while non-ulcerated lesions are in general thinner. Mainly for these reasons, but with the additional evidence that a sex–ulceration interation with respect to prognosis existed ($P<0.0001$), it was important to consider the four distinct subgroups of sex and ulceration status – males and females with ulcerated or non-ulcerated lesions.

Stepwise proportional hazards models were then fitted to the data from each of these four subgroups (Table II). It will be seen that within these four subgroups the choice and effects of the remaining significant prognostic factors are very different. This is particularly apparent for tumour thickness.

**Table I** Significance of potential prognostic factors

| Factor | *Individual significance on full survival data* $\chi^2$ *(d.f.)*[a] | $P^a$ | *Order of inclusion in stepwise PH*[b] *model* Order | $\chi^2$ *(d.f.)*[b] | $P^b$ |
|---|---|---|---|---|---|
| Ulceration (present/absent) | 188 (1) | <0.01 | *1* | *188 (1)* | *<0.01* |
| Tumour thickness | 136 (1) | <0.01 | *2* | *52 (1)* | *<0.01* |
| Mitosis (low/medium/high)[c] | 100 (2) | <0.01 | 7 | 8 (2) | 0.02 |
| Level of invasion (Clark level 2, 3, 4, 5) | 98 (3) | <0.01 | 6 | 15 (3) | <0.01 |
| Histogenetic type[d] | 93 (4) | <0.01 | 8 | 4 (1) | 0.05 |
| Age | 45 (1) | <0.01 | 4 | 13 (1) | <0.01 |
| Sex (male/female) | 33 (1) | <0.01 | *3* | *22 (1)* | *<0.01* |
| Anatomical site (head and neck, trunk, upper limbs and lower limbs) | 24 (3) | <0.01 | 5 | 15 (3) | <0.01 |
| Pre-existing naevus (no/yes) | 4 (1) | <0.05 | Not included | | |
| Regression associated with vertical growth phase (no/yes) | 2 (1) | 0.15 | Not included | | |
| Regression associated with horizontal growth phase (no/yes) | 0.5 (1) | 0.50 | Not included | | |

[a]Chi-squared values, degrees of freedom and $P$-values for tests of significance of *individual* factor. [b]As above but for the *inclusion step* of the factor in a forward stepwise proportional hazards (PH) model. [c]Levels of mitosis are: low = <1 per 10 h.p.f, medium = 1–5 per 10 h.p.f, high = >5 per 10 h.p.f. [d]Histogenetic types are: lentigo maligna, superficial spreading, nodular, acral and others. In the stepwise approach only the acral group showed a significant difference among the types.

**Table II** Significance of prognostic factors for sex/ulceration subgroups

| Subgroup | No. of complete cases | Per cent alive after 5 years | Significant risk factors for stepwise proportional hazards model: Risk factors | $\chi^2$ (d.f.)[a] | P[a] |
|---|---|---|---|---|---|
| Females with ulceration | 269 | 53% | Tumour thickness | 13 (1) | <0.01 |
| | | | Mitosis[b] | 10 (1) | <0.01 |
| | | | Anatomical site[b] | 10 (1) | <0.01 |
| Females without ulceration | 714 | 90% | Tumour thickness | 53 (1) | <0.01 |
| | | | No other factors included | | |
| Males with ulceration | 137 | 35% | Level of invasion[c] | 5 (1) | 0.03 |
| | | | No other factors included | | |
| Males without ulceration | 280 | 78% | Tumour thickness | 33 (1) | <0.01 |
| | | | Level of invasion[d] | 8 (1) | <0.01 |

[a]Chi-square values, degrees of freedom and *P*-values for test of significance of the prognostic risk factor when entered into the proportional hazards model. The factors are given in order of entry to the model. [b]Here, after appropriate tests, mitosis was coded as low *vs* medium/high while anatomical site was coded as axial *vs* extremity. [c]Here, after appropriate tests, level of invasion was coded as levels 3 and 4 *vs* 5. [d]Here, after appropriate tests, levels of invasion was coded as levels 4 and 5 *vs* the other levels.

**Table III** Summary of the performance of the survival analysis prognostic procedure on the SMG 1987 data

| 1987 Patient subgroup | Number of years after diagnosis: 1 | 2 | 3 | 4 |
|---|---|---|---|---|
| *Females with ulcerated lesions* | | | | |
| No. of patients at risk | 46 | 44 | 44 | 33 |
| Observed/predicted ratio[a] | 42:42.4 | 36:33.9 | 33:30.3 | 21:19.8 |
| *Females with non-ulcerated lesions* | | | | |
| No. of patients at risk | 151 | 149 | 143 | 132 |
| Observed/predicted ratio[a] | 150:149.9 | 148:146.5 | 141:139.5 | 129:126.8 |
| *Males with ulcerated lesions* | | | | |
| No. of patients at risk | 32 | 30 | 30 | 28 |
| Observed/predicted ratio[a] | 28:29.1 | 20:22.1 | 18:18.5 | 15:13.7 |
| *Males with non-ulcerated lesions* | | | | |
| No. of patients at risk | 71 | 68 | 66 | 61 |
| Observed/predicted ratio[a] | 71:69.2 | 65:63.3 | 61:59.8 | 54:51.8 |

[a]Ratio of observed patients to those predicted to survive beyond this time. Observed numbers are those whose actual status (i.e. dead due to melanoma or not) was certain the appropriate number of years after prognosis.

**Table IV** Predicted prognosis for groups of patients classified according to proportional hazards model

| Patient subgroup: Sex | Ulceration | Other factors | n | Five year survival at three tumour thicknesses: 1 mm | 3 mm | 5 mm |
|---|---|---|---|---|---|---|
| Female | Without | – | 855 | 95% (93, 96) | 90% (88,93) | 82% (77, 87) |
| Male | Without | Level of invasion 2 or 3 | 187 | 93% (90,98) | NA | NA |
| Male | Without | Level of invasion 4 or 5 | 157 | 81% (75, 89) | 72% (64, 80) | 59% (50,70) |
| Female | With | Extremity site few mitoses | 83 | 81% (70, 94) | 80% (72, 90) | 79% (69, 91) |
| Female | With | Axial site few mitoses | 43 | 75% (60, 94) | 67% (51, 87) | 56% (39, 79) |
| Female | With | Extremity site many mitoses | 143 | 62% (53, 72) | 59% (51, 69) | 57% (49, 66) |
| Female | With | Axial site many mitoses | 60 | NA | 62% (48, 81) | 53% (40, 72) |
| Male | With | Level of invasion 3 or 4 | 154 | 53% (43, 66) | 49% (41, 59) | 45% (37, 54) |
| Male | With | Level of invasion 5 | 32 | NA | NA | 37% (20, 69) |

NA, not enough data to produce reasonable prediction. Numbers in brackets are approximate 95% confidence intervals.

Females with ulcerated lesions have three further features of prognostic significance: tumour thickness, mitotic count in tumour cells and anatomical site of primary tumour. If the primary tumour is not ulcerated, tumour thickness is a very important prognostic variable in both sexes, but its value is greatly diluted in the presence of ulceration, particularly for males. For example males with ulcerated primary tumours have a less than 50% chance of surviving 5 years irrespective of level of invasion, but for other subgroups survival prospects beyond 5 years are better.

The proportional hazards model based on these subgroups and derived from patients diagnosed between 1979 and 1986 inclusive was then applied to patients first diagnosed in Scotland in 1987. For each patient in this validation sample, a prediction of survival status was estimated on the basis of the 1979–86 derived model and compared with actual status at 1, 2, 3 and 4 years' follow-up. The results are summarised in Table III, from which it will be seen that there is excellent agreement between predicted and actual total numbers of survivors at the corresponding timepoints.

Based on the PH models for each distinct subgroup (Table II), 5 year survival probabilities were calculated to illustrate prognosis for patients with melanomas 1 mm, 3 mm and 5 mm thick. This is shown in Table IV, from which it will be seen that survival prospects vary greatly depending on the sex of the patient and whether or not the lesion is ulcerated. For example, melanomas of 1 mm are generally considered to have a good prognosis, and this is true for non-ulcerated melanomas in women with 94% 5 year disease-free survival; however, for a male with 1 mm lesion which is ulcerated and has invaded to Clark level 3 or 4, 5 year survival prospects are only 51%. Conversely, a primary melanoma 5 mm thick is considered as a poor prognosis lesion but, if occurring in a female and non-ulcerated, 5 year disease-free survival prospects are 82% for a tumour of the same thickness. These figures make the point that for adequate counselling of patients and their families, more information than just tumour thickness must be considered. Table IV also makes the point that risk of recurrence is far higher in ulcerated lesions, particularly in males, and that it might therefore be prudent to follow up males at 3 monthly intervals for the first 5 years after surgery, in the hope of detecting recurrences at a very early stage when further surgical intervention is more likely to prolong survival.

### Discussion

The aim of this study has been to use sophisticated statistical techniques on a large geographically based database to obtain prognostic information relevant to that population, and to identify subsets of patients with markedly different survival prospects. All such models require validation, and ours has been validated within the same geographic area with an independent patient population not used for the original derivation of the model. Validation using a melanoma patient group drawn from a different geographic area is now required to test the geographic universality of the model. This is important, as in our experience the models derived by Clark *et al.* (1989) and by Soong *et al.* (1992) do not accurately reflect the situation in Scotland. Not only do the estimated survival prospects differ markedly in many of the subgroups considered in these models, but the choice of important prognostic features is substantially different. For example, for tumours 0.76–1.49 mm thick the Soong model incorporates anatomical site, ulceration and level of invasion as the important and essential prognostic features, whereas exactly the same analysis on that subgroup for the Scottish Melanoma Group database throws up a completely different set of essential features, i.e. sex, age and regression in the horizontal growth phase. However, even if the Soong *et al.* model is applied, differences of up to 30% in 5 year survival prospects were discovered between the Sydney/Alabama and Scottish databases in other subgroups defined by the Soong *et al.* choice of prognostic features.

Further experience emphasises the fact that carrying out logistic regression to a fixed time point such as 8 years as in the Clark model can seriously bias results if a large number of cases are lost over time to follow-up. Omitting these cases could underestimate survival prospects by as much as 10%.

This study emphasises the importance of using appropriate statistical analyses to study the interaction between factors of suspected prognostic significance. The differing results in the literature may well be due at least in part to a failure to study these interactions fully, by only considering features of putative prognostic significance in isolation. However, there may also be geographic variation in prognostic factors, based for example on subtle effects on the immune system caused by intense UV exposure in high melanoma incidence countries such as Australia. It is likely that in different parts of the world where median tumour thicknesses differ, and the incidence of melanoma is significantly different, these prognostic models may be less relevant. Accordingly, each large centre may need to derive its own model, i.e. set of relevant prognostic features and their interactions, from retrospective data on which 5 year follow up at minimum is available, and then validate it on a second data set from that area. We would, however, anticipate that the model presented here will be relevant at least to a northern European setting.

**Acknowledgements**
We are grateful to the Scottish Hospital Endowment Research Trust for financial support and to the Cancer Research Campaign for funding the Scottish Melanoma Group. We also wish to thank the Scottish Melanoma Group secretaries Miss E Salt and Mrs J Stewart for their help with this project.

### References

BALCH CM, SOONG SJ, SHAW HM, URIST MM AND McCARTHY WH. (1992). An analysis of prognostic factors in 8500 patients with cutaneous melanoma. *Cutaneous Melanoma*, 2nd edn, pp. 165–187. Lipincott: Philadelphia.

BRESLOW A. (1970). Thickness, cross sectional area and depth of invasion in the prognosis of cutaneous melanoma. *Ann. Surg.*, **172**, 902–904.

BRESLOW A. (1975). Tumour thickness, level of invasions and node dissection in stage 1 cutaneous melanoma. *Ann. Surg.*, **182**, 572–576.

CLARK WH, ELDER DE, GUERRY D, BRAITMAN LE, TROCK BJ, SCHULTZ D, SYNNESTVEDT M AND HALPERN AC. (1989). Model predicting survival in stage 1 melanoma based on tumour progression. *J. Natl Cancer Inst.*, **81**, 1893–1904.

EVERITT BS. (1989). *Statistical Methods for Medical Investigation*, pp. 83–98. Oxford University Press: New York.

MACKIE RM, HUNTER JAA, AITCHISON TC, HOLE D, McLAREN K, RANKIN R, BLESSING K, EVANS AT, HUTCHEON AW, JONES DH, SOUTAR DS, WATSON ACH, CORNBLEET MA AND SMYTH JF. (1992). Cutaneous malignant melanoma in Scotland 1979–1989. *Lancet*, **339**, 971–976.

MACLENNAN R, GREEN AC, MCLEOD GRC AND MARTIN NG. (1992). Increasing incidence of cutaneous melanoma in Queensland Australia. *J. Natl Cancer Inst.*, **84**, 1427–1432.

SOONG SJ, SHAW HM, BALCH CM, McCARTHY WH, URIST MM AND LEE JY. (1992). Predicting recurrence and survival in localised melanoma: a multivariate approach. *World J. Surg.*, **16**, 191–195.

VOLLMER, RT. (1989). Malignant melanoma: a multivariate analysis of prognostic factors. *Pathol. Annu.*, **24**, 383–407.